# Assessment as Learning: How Does Peer Assessment Function in Students' Learning?

**DOI:** 10.3389/fpsyg.2022.912568

**Published:** 2022-06-27

**Authors:** Shengkai Yin, Fang Chen, Hui Chang

**Affiliations:** ^1^School of Foreign Languages, Shanghai Jiao Tong University, Shanghai, China; ^2^School of Languages and Linguistics, The University of Melbourne, Parkville, VIC, Australia

**Keywords:** peer assessment, self-assessment, formative assessment, assessment as learning, Many-Facet Rasch Measurement, language learning, self-regulated learning

## Abstract

Peer assessment (PA) is employed as one fundamental practice of classroom-based assessment in terms of its learning-oriented and formative nature. The exercise of peer assessment has multiple and additional benefits for student learning. However, research into the learning processes in peer assessment is scarce both in theory and in practice, making it difficult to evaluate and pinpoint its value as a tool in assessment as learning (AaL). This study focuses both on the learning process and outcome through assessment activities. We set out with three goals in mind: (1) to examine students' assessment performance in context, (2) to evaluate its impact on student progress, and (3) to illuminate teachers on organizing assessment activities. Three specific research questions are answered in this study: (1) How do student raters perform in the process of PA in an advanced English class? (2) To what extent do assessment activities influence the students' speaking ability? (3) What are students' perceptions of PA regarding its benefits and caveats? A total of 29 undergraduate students participated in two assessment activities on argumentative speaking. Many-Facet Rasch Model analysis was conducted to measure the rater effects both at the group level and the individual level. Bias/interaction analyses were performed to diagnose rater behavior in different contexts including the rating session, speaking session, and peer assessment vs. self-assessment. Questionnaire and semi-structured interview data were also collected to explore factors and strategies that could interfere with PA as AaL. Results show that students exhibited stable rating behavior and made progress in argumentative speaking in all dimensions, including delivery, organization, and language use. They are more stringent with themselves than with peers although there is one rare case with a bias against peers. Participants acknowledged the benefits of PA but also shared reasonable concerns in practice. This study validated the feasibility and the effectiveness of PA for student learning. Discussion on findings and guidelines for effective implementation of PA as AaL are provided.

## Introduction

Classroom assessment has shifted from being a tool to record student achievement to an activity to support learning. A body of research has emerged by looking at how such a paradigm can best serve the learning process (Turner, 2012; Turner and Purpura, 2016). As one of the three main classroom assessment approaches, assessment as learning (henceforth, AaL) is different from assessment of learning (henceforth, AoL), and assessment for learning (henceforth, AfL). It is acknowledged that AoL functions as a summary of learning and AfL aims at enhancing learning and teaching from teachers' perspectives (Black and Wiliam, 2009). Shifting from the emphasis on teachers, AaL focuses on individual student's active role in the assessment process and thus has received considerable attention in the field of the second language (L2) learning and teaching (Earl, 2013; Lee, 2016). Several studies have also identified the potential benefits of AaL in fostering students' metacognitive awareness (Xiang et al., 2021), self-reflection (Lam, 2016), and accountability for their learning (Rourke, 2012). All these are broadly applicable to successful L2 learning.

Peer assessment (henceforth, PA) is one of the most common AaL strategies that has been widely employed in classroom settings (Yan and Boud, 2022). However, there is a dearth of studies describing how PA can be implemented in the AaL-focused L2 learning context (To and Panadero, 2019; Xiao and Gu, 2022). What complicated the scenario is that such limited number of expositions are unsatisfactory because of their limited methods of data collection and little discussion about the assessment and measurement aspect *per se*, which should have been the foundation as AaL's name suggests.

This study will explore both the learning process and the assessment outcome in peer assessment in the class as a form of AaL. We will focus on speaking ability since this topic received scant attention in AaL studies. We set out with three specific goals in mind: (1) to describe how raters behave as they assess peers, (2) to compare the score variations of examinees in different contexts, and (3) to illuminate teachers on how to arrange peer-assessment activities.

## Literature Review

### Formative Assessment vs. Summative Assessment

The last several decades have witnessed a strong interest in formative assessment, which is designed to offer a wealth of feedback and support for learning (Black and Wiliam, 1998). Different from summative assessment which features neutral methods and tools for measuring the outcome, formative assessment is a process and function involving multiple components that interact and shape student growth (Wiliam, 2011). Typical differences between these two lie in the assessment components, including test purpose, focus, examiner-to-examinee relationship, and feedback to students (see Table 1). These components tend to be negotiated as local decisions but they form a chain of evidence that works in synergy to encourage student development.

**Table 1:** The differences between formative assessment and summative assessment.

	**Formative assessment**	**Summative assessment**
Purpose	Use the information to adjust teaching and learning to meet student needs	Provide information to judge the overall value of an educational program
Focus	The process of learning	The outcome of learning
Examiner/examinee relationship	Examiners can intervene in the assessment process in an effort to teach and help students	Examiners are expected to adopt a neutral and disinterested stance as a means of minimizing measurement error
Feedback	Given during the process of assessment and can take a variety of forms	Little or no feedback is given on the quality of performance until the assessment is complete

There are three main stakeholders (i.e., agents) in formative assessment, including teachers, learners, and peers. Teachers need to help students understand the criteria for success and present evaluative opportunities to the latter by conducting assessments. Students should be responsible for their learning and make adjustments by asking questions, doing self-reflection, and revising their work in response to the assessment results (Turner and Purpura, 2016). At least three types of dyadic relationships are relevant in this process, including examiner-to-examinee, examinee-and-self, and examinee-to-examinee. However, the dynamics between human beings can be rather complicated, which adds to the challenges for effective formative assessment (Bennett, 2011).

### Assessment as Learning

#### Disentanglement of Assessment of, for, and as Learning

In a critical review analysis, Zeng et al. (2018) distinguished three functions of formative assessment: assessment *of* learning (AoL), assessment *for* learning (AfL), and assessment *as* learning (AaL). All of these are considered critical reasons for the current trend favoring formative assessment in the classroom.

AoL is typically administered at the end of a course or unit of instruction. The purpose is to assess how desirably instructional goals have been achieved and to quantify student achievement or grant certification as a result of learning (Linn and Gronlund, 2000). Standardized testing, such as high school graduation examinations, is one typical example of AoL where objective items are usually used to assess the general abilities of students to ensure fairness and justice. AoL can occur in the process of FA, but mainly to generate evidence about learner differences.

AfL is an assessment activity that can support learning by providing information that teachers and students can use in evaluating themselves and each other. It is designed to make each student's understanding visible so that teachers can adjust their teaching to help students to progress (Black et al., 2004). The purpose of AfL is to pinpoint the current location of learners along the learning process, find out what they need, and decide on the best approach to help them get to the destination (Broadfoot et al., 2002). Useful information and data can be collected by the assessment to inform subsequent teaching and learning. However, teachers are the center of the process of AfL rather than students. Thus, AfL is formative more for the teacher rather than for the students (Black et al., 2003).

AaL refers to students' active involvement in their assessment, treating assessment as a learning process (Zeng et al., 2018). AaL stems from the idea that learning is not merely about transferring information from a knowledgeable person to a rookie learner, but an active process where learners are engaged with continuous assessment of knowledge needs and learn to re-construct relevant cognitive understanding in context. More importantly, they learn to internalize assessing-to-learn as a mental habit (Earl, 2006). Zeng et al. (2018) summarized three fundamental features of AaL including the assessment role for metacognitive processing, students' external role as the critical connector between assessment and learning, and students' internal role as a self-regulator to achieve their learning goal. AaL is of paramount value to students because it requires students to be responsible for their learning (Andrade, 2010; Hall and Goetz, 2013), which has long been acknowledged to yield greater academic success for students in and beyond school (Lau, 2013).

Previous attempts to conceptualize AaL have offered diverse perspectives on the “learning” aspect of the concept, but still, relatively few studies touch upon the “assessment” aspect *per se*. For example, Li (2018) investigated the validity and washback issue of using self-assessment (one form of AaL) in the context of translator and interpreter education. They provided evidence of criterion-related validity to examine the measurement aspect of self-assessment; however, little was explained on the rating process, which should be key validity evidence for score interpretation.

Yan and Boud (2022) balanced the relationship between assessment and learning and defined AaL as “assessment that necessarily generates learning opportunities for students through their active engagement in seeking, interrelating, and using evidence” (p. 13). This definition is distinctive from others because it focuses on the essence of AaL in enacting a learning strategy through assessment while acknowledging the students' active and responsible role in the whole process. As indicated in other studies, such engagement with tasks and activities can lead to students' development in metacognition and self-regulation (Earl, 2006, 2013; Clark, 2012; Dann, 2012). A full elaboration on metacognition and self-regulation is beyond the scope of this article; however, in brief, metacognition refers to an individual's knowledge of one's cognitive phenomena (Flavell, 1979), while self-regulation is the process that influences the external environment by self-observation, self-judgment, and self-reaction (Bandura, 1986). Or, metacognition helps to consciously control and monitor the thoughts, while self-regulation serves to translate the thoughts into behaviors. Together, they form the essence of AaL.

Instead of taking assessment as a summary of learning, AaL takes a further step in emphasizing the importance of assessment activities that maximize the learning opportunities and enhance student responsibility in the assessment process (Yan and Yang, 2022). In AaL, more learning happens when students are involved in completing the tasks and reflecting upon what they can do (Boud, 2022).

#### What Does AaL Have to Offer for Language Assessment and Learning?

As mentioned above, metacognitive knowledge development is notably crucial to university students for academic success (Pintrich and De Groot, 1990; Pintrich, 2002). Recent research in English education has also prioritized AaL for the exact reason (Lam, 2016; Xiang et al., 2021; Wang and Xu, 2022). AaL in the English classes has been explored both from the student's perspective and the teacher's perspective. For example, Xiang et al. (2021) demonstrate that AaL strategies (e.g., peer assessment, self-assessment, giving feedback, making revisions, etc.) helped students develop their metacognitive awareness and resulted in enhanced assessment and feedback literacy. Wang and Xu (2022) explored how the implementation of AaL design by two different teachers interplayed with metacognition but both drew out the positive disposition of college-level English learners.

The core foundation of AaL lies in students acting as critical connectors, active thinkers, and knowledge contributors (Earl, 2013). Similarly, in applying AaL in the English as a Foreign Language (EFL) context, learners are regarded as active agents who are encouraged to become their assessors during the language assessment and learning process (Lam, 2016).

A great body of research has demonstrated the positive role of AaL in language learning through students' agentic engagement (Fletcher, 2016, 2018; Wang and Lee, 2021). For example, Wang and Lee (2021) investigated three Chinese undergraduates engaged in L2 writing assessment in the classroom. They conceptualized student engagement in AaL-focused writing class as the fourth and core dimension in the engagement process, together with emotion, cognition, and behavior engagement. Through multiple data sources, they found that students represented different degrees of engagement, which boiled down to reciprocal learning in the assessment context and proactive self-regulation of learning by the students. The study underscores students' agent role in L2 writing assessment and provides insights for university English teachers on how teaching and assessment methods can encourage students' active participation.

In addition, AaL can serve as a crucial medium in the language classroom to foster students' evaluative judgment either on the quality of one's own or others' work (Tai et al., 2018). Boud et al. (2018) summarized five components of evaluative judgment, which were matched with the corresponding features of AaL in a later study (Boud, 2022). The components encompass discerning quality, judgment processes, managing biases, assessing the trustworthiness of sources and others, and seeking practice opportunities. Following the same lead, Xiao and Gu (2022) conducted a qualitative analysis on how AaL fostered the development of evaluative judgment in an English-for-academic-purposes class. The results showed that peer assessment and self-assessment practices provided students with valuable experiences by offering them opportunities to compare their work with others', understanding the standards more clearly and identifying the strengths and weakness of themselves.

### Peer Assessment as AaL

Peer assessment (PA) and self-assessment (SA) are two common types of AaL strategies (Yan and Boud, 2022). It has been widely discussed and has been found to promote student-centered education (Tai and Sevenhuysen, 2018; Xiang et al., 2021).

Topping (1998) defined PA as “an arrangement in which individuals consider the amount, level, value, worth, quality, or success of the products or outcomes of learning of peers of similar status” (p. 250). In 2009, he detailed the products to be assessed, which included written work, oral presentations, portfolios, test performance, or other skilled behaviors (Topping, 2009). All these products are regarded as inherent components of classroom activities, which indicates his theoretical reconceptualization in favor of the formative feature of PA.

In the EFL context, the exercise of PA has additional benefits, including improvements in the effectiveness, motivation, and quality of learning (Zhao, 2010; Shih, 2011; Adachi et al., 2018), and increased student responsibility and autonomy (Shen et al., 2020). PA can also improve students' self-assessment by expanding their understanding of quality, judgment of performance, and encouraging self-reflection on strengths and weaknesses (To and Panadero, 2019).

PA has been applied to the language classrooms and has claimed to share the features of AaL. For example, Shen et al. (2020) explored the effects of peer assessment on learner autonomy. The one-semester-long intervention study involved seventy English major sophomores in a college English writing class in China, with one control group using traditional teacher feedback and one experimental group adopting peer assessment. Results showed that peer assessment enhanced learner autonomy in terms of a changed view on the expected role of the teacher, reduced dependency, and boosted student confidence in study ability, leading to an elevated agency.

However, most PA studies centered on writing, and relatively few explored the speaking mode which is another important productive skill that merits more consideration (Aryadoust, 2015). Besides, the majority of the studies examined the students' perceptions *after* the instruction; few tracked the student engagement throughout the AaL activities over time or probed into the assessment process (Stančić, 2021). Limitations in sample size (To and Panadero, 2019; Tsunemoto et al., 2021) or uni-dimensional qualitative approach also restricted the generalizability of findings to other populations and contexts.

### Research Questions

As revealed in the literature, a major concern looming large over AaL research is that few of them touched upon the assessment part in AaL, which includes the students' task performance, measurement bias, the construct being assessed, and the reliability and validity of the rating processes (Aryadoust, 2016). Without direct evidence of these processes, implications for pedagogy and learning would be groundless.

This study is therefore designed to add one concrete example to explore the working processes of AaL in the classroom, especially PA as a form of AaL in college-level EFL classes. We will focus on peer assessment of speaking performance and plan to investigate to what extent peer assessment influences students' learning. This study will answer the following three questions:

Research question 1: How do student raters perform in the process of peer assessment?

Research question 2: To what extent do peer and self-assessment activities influence students' speaking ability?

Research question 3: What are students' perceptions of peer assessment regarding its merits and caveats?

## Materials and Methods

### Participants and Instruments

A total of twenty-nine Chinese-speaking English learners (16 males and 13 females) and two faculties participated in this study. All the English learners were freshmen in a top university in China with various majors. The two faculties were the classroom teacher and teaching assistant and they were also researchers in this study. All students responded to two rounds of argumentative speaking tasks and they also rated their performance and their peers'.

The augmentative speaking tasks (Appendix A in Supplementary Material) were jointly developed by three researchers in this study, all of whom had experience teaching English as a foreign language or designing language assessment tasks. There were four questions and students were randomly assigned one as the first speaking task and a different one as the second task. Three scores were awarded analytically on three standards, including delivery, language use, and organization. A rating scale from 1 to 5 (1 being the lowest and 5 the highest) was adopted using TOEFL iBT independent speaking rubrics descriptors (https://www.ets.org/s/toefl/pdf/toefl_speaking_rubrics.pdf).

### Data Collection Procedures

**Figure 1:**
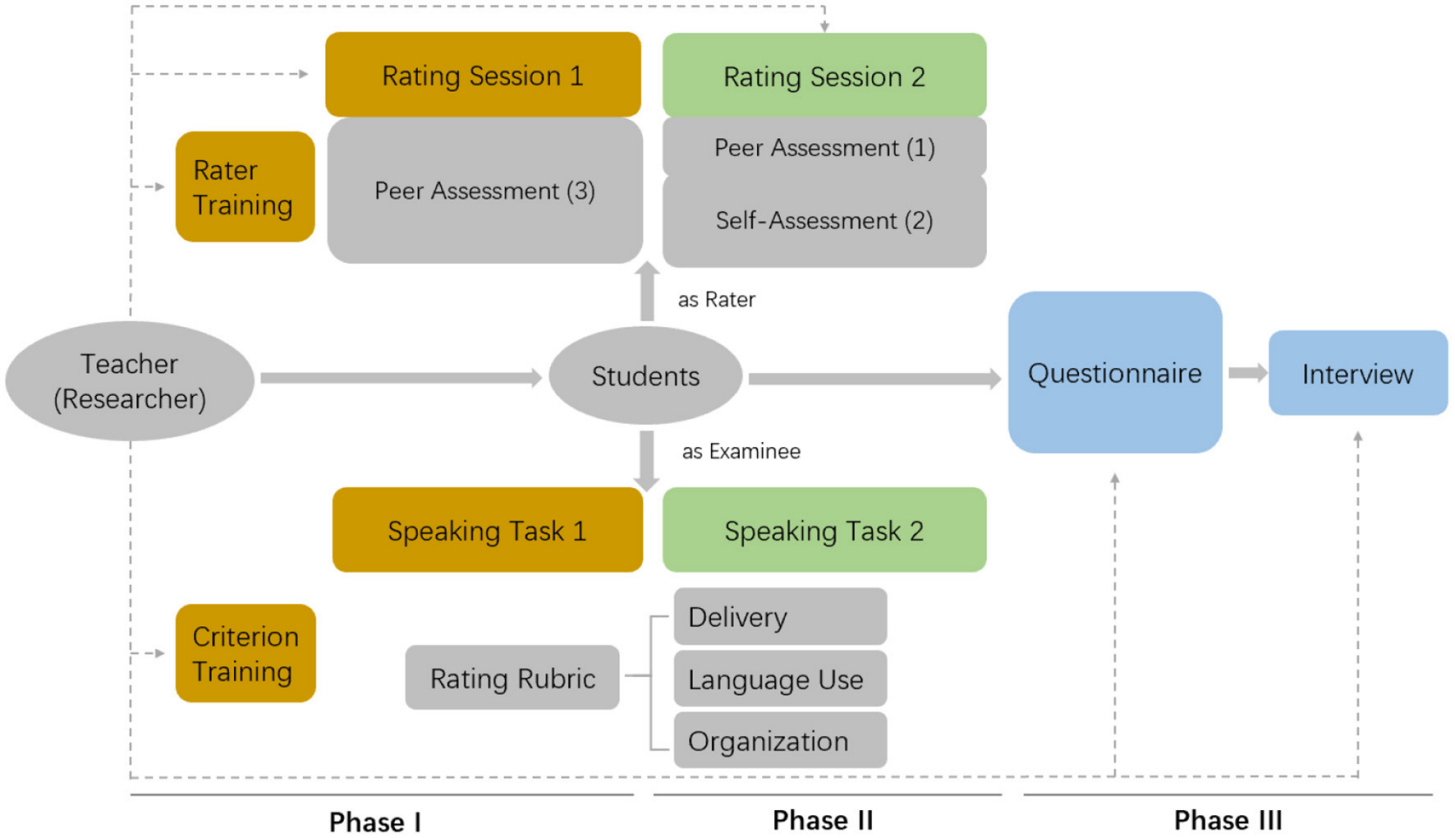
Flowchart of the research procedure with an incomplete but linked data collection design.

We adopted an explanatory sequential mixed methods research design (Creswell and Plano Clark, 2018). We collected quantitative data first, analyzed the results, and then used the results to build the qualitative part. Quantitative and qualitative results were complemented, compared, and contrasted in a joint display. Specifically, this research consisted of three phases. The phases and their component tasks were linked through an overall design that controlled for possible confounding effects explained below (Figure 1).

In Phase I, students responded to one argumentative speaking task and assessed three peer recordings. Before assessing, a brief in-class training on peer assessment was conducted. Students learned about the standards for good speaking and were given chances to practice awarding scores. After that, the first assessment task was given. Students had a whole week to evaluate three peer recordings according to the rubrics. This was a double-blind process where raters and recordings were assigned different anonymous IDs known only to the student rater and the author of the recording.

In Phase II, students responded to a second speaking task. They then assessed one peer recording for the same question they were assigned as well as their responses to the two speaking questions in Phase I and Phase II. Thus, this Phase involved both a self-assessment (SA) and a peer-assessment (PA) activity. In this Phase, students were also asked to provide written justifications for PA rating. All identifiable personal information was removed from the feedback which was then sent to the corresponding student author through the online instructional platform provided by the university.

The whole research was designed in such a way that multiple links were established between peer assessment and self-assessment and between different rating sessions through common recordings and common raters. The measurement errors due to relevant facets were thus controlled simultaneously to support valid comparison and interpretation of results. For example, if a student was assigned question A as the speaking task in Phase I, he or she would be required to rate a peer response to question A in addition to two other responses to questions B and C. Then, in Phase II, the student would be assigned question D as the second speaking task so that he or she had chances to learn from peers but no chance to copy verbatim from others. For the assessment task in Phase II, the student would be assigned a peer recording on question D to rate as well as to self-rate his or her responses to question A from Phase I and question D from Phase II.

In Phase I and Phase II, students were given different rater and recording IDs to ensure anonymity and discourage any possible peer or social influence for unfair ratings. A faculty rating was also given to all recordings to provide a criterion for checking student rater behavior and serve as an additional link among various facets. On average, about 7 to 8 students were assigned the same speaking task each time, and every recording was rated by an average of 2–4 students (one being themselves) plus a researcher. This way, differences in rater leniency and task difficulties were controlled by design. To maximize learning opportunity, adjusted rating scores based on the Many-Facet Rasch Measurement (MFRM) model and descriptive statistics of the rating and rater leniency distribution were shared in class after the quantitative analyses were done.

In Phase III, all the participants (*N* = 29) filled out a paper-based questionnaire where they provided their background information and reflected on their rating experience. They reported their gender, specialties, and previous PA rating experience. They, then, rated the extent to which they agreed or disagreed with certain statements regarding their attitude toward the assessment activities, their rating process, and opinions on the assessment effects. The questionnaire used a 5-point Likert scale ranging from “strongly disagree” to “strongly agree” for each descriptor. Appendix B in Supplementary Material shows all the questions included in the questionnaire. The first two authors conducted face-to-face semi-structured interviews to triangulate the obtained questionnaire data and the MFRM results (Brinkmann, 2013). Four students (two males and two females) were invited for the interview in the Chinese language. The selection of participants took into consideration of students' willingness to participate, their gender, major, and speaking performance, to achieve maximum variation in the sampling. All interviews were administered after the course was over and the final grades were officially posted and they were conducted either in the classroom or office on campus. Thus, the participants could feel at ease articulating their genuine thoughts and ideas (Dörnyei, 2007). All the interviewees signed a consent form before being interviewed and audio-recorded. Each interview lasted for about 20 min. The 32-item Consolidated Criteria for Reporting Qualitative Research checklist was used to ensure the transparency and quality of the research (Tong et al., 2007).

### Measurement Models and Evaluation Criterion

#### Many-Facet Rasch Measurement

The rating data were analyzed using Many-Facet Rasch Measurement (MFRM) with the software FACETS (Version No.3.82.2) (Linacre, 2018b). MFRM is an analytic approach in the rater-mediated assessment that allows for fine-grained evaluation of the rating behavior of individual raters and estimation of student proficiency while adjusting for differences in rater variability (Styck et al., 2021). It also allows examination of other aspects of the rating scenario, such as the rubric, and interaction between factors (Myford and Wolfe, 2003, 2004). Overall, this model is one of the most useful validation tools for identifying and quantifying the impacts of facets in performance assessments (e.g., Eckes, 2009; Matsuno, 2009), and it is applicable in this study for evaluating the rater's behavior and the assessment process.

This study involves a six-facet model, including student-as-rater severity (Rater), student-as-examinee's ability (Examinee), task session (TaskSession), rating session (RateSession), rater status (SelfPeer), and standards (Standard). The model can be expressed as:


$$\begin{array}{l} ln\left[P_{\text{nijklmq}}/P_{\text{nijklmq}-1}\right]=B_{\text{n}}-D_{\text{i}}-C_{\text{j}}-E_{\text{k}}-F_{\text{l}}-G_{\text{m}}-H_{\text{q}} \end{array}$$

where

P_nijklmq_ = probability of examinee *n* being rated *q* on trait *i* by rater *m* for task session *j*, rating session *k*, and rater status *l*,

P_nijklmq−1_ = probability of examinee *n* being rated *q-1* on trait *i* by rater *m* for task session *j*, rating session *k*, and rater status *l*,

B_n_ = level of performance for examinee n,

D_i_ = difficulty of standard *i*,

C_j_ = difficulty of task session *j*

E_k_= difficulty of rating session *k*

F_l_ = difficulty of rater status *l*

G_m_ = severity of rater *m*

H_q_ = difficulty of scale category *q* relative to scale category *q-1*

Model fit and person fit were evaluated according to Linacre (2012), for example, Infit statistics below 0.5 was considered overfit, those between 0.5 and 1.5 acceptable fit, and those over 1.5 misfit. Overfit means rater performance is too predictable than the Rasch model predicts and misfit denotes that the raters are unpredictable (Myford and Wolfe, 2003, 2004). Linacre (2018b) also suggested that satisfactory global model-data fit is achieved when ≤5% of standardized residuals with absolute values are greater than 2 and ≤1% of those are greater than 3.

#### Questionnaire and Interview

The questionnaire and interview were conducted for triangulation purposes. Items in the questionnaire were summarized with descriptive statistics (Appendix B in Supplementary Material). The interview followed a similar structure to the questionnaire but included on-the-fly exploration for deeper and nuanced understanding. The interview transcript was repeatedly read and analyzed to discover the emerging themes. NVivo software for Windows (version 12 plus) was employed for coding. All researchers discussed the disparities and reached an agreement on a final coding to ensure inter-coder reliability. Four main themes were settled after rounds of iterations and revisions of the coding scheme (see Appendix C in Supplementary Material).

## Results

To evaluate PA as an AaL, both the assessing process and the assessment outcomes were examined. In the following sections, we will first present general results on all the six facets, then explore the diagnostic details to answer the specific research questions.

### General Distribution of Measurement Results

**Figure 2:**
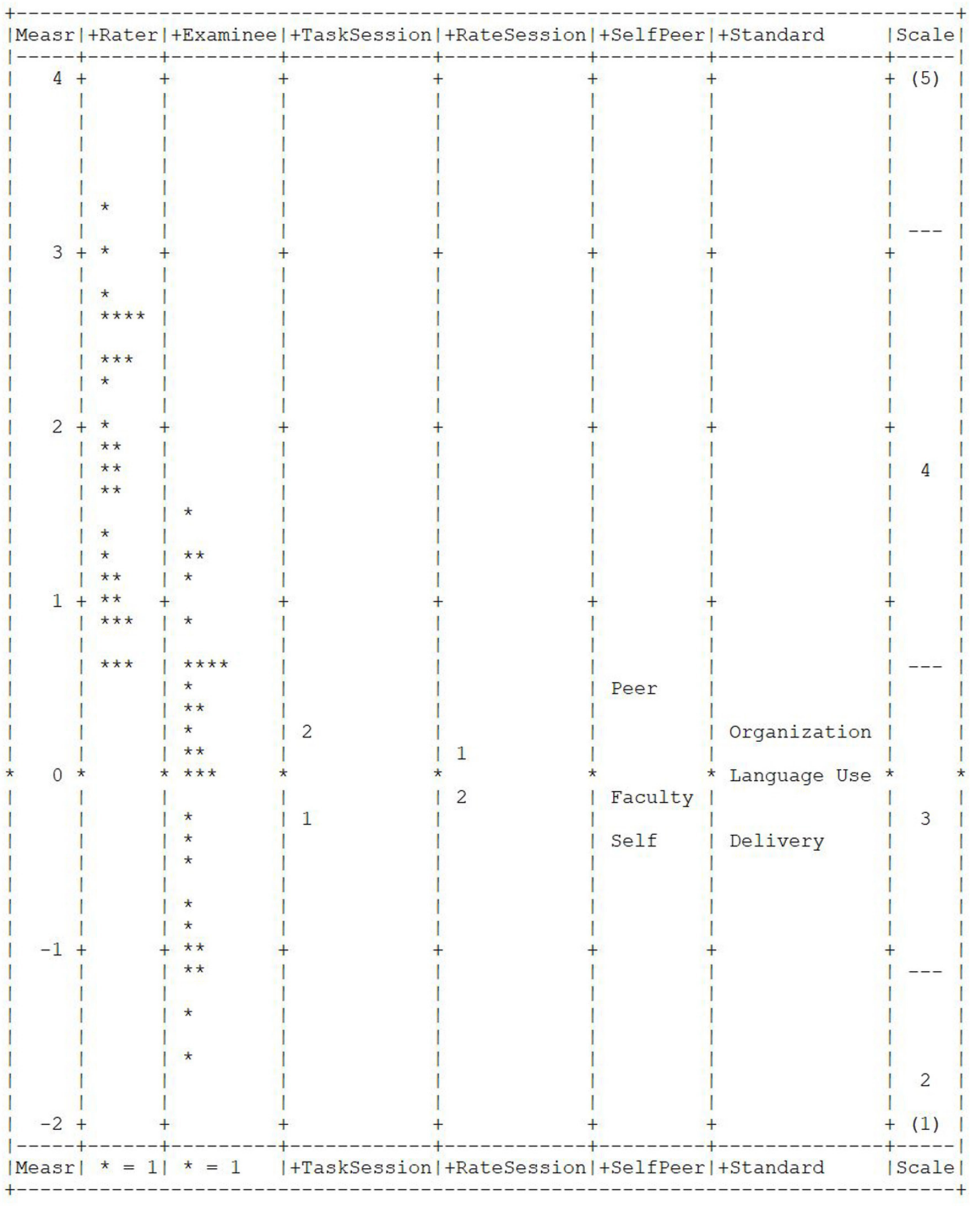
Wright map of all facets.

Wright map from MFRM analyses presented the relationship between all facets (Figure 2). The measurement “ruler” (column 1) spanned from −2 to 4 with specific estimates between −1.67 and 3.26. There was no obvious outlier in any facet. Rater scale (column 2) spread as widely as Examinee (column 3), with the average leniency around 1.76, while the mean for examinee abilities was 0 (Table 2). This indicated that raters were rather lenient in awarding the scores compared to the actual quality of the products. The second speaking task received higher scores and this difference reached statistical significance with a fixed χ^2^ of 20.1 (*p* = 0.00). Thus, student performance on argumentative speaking improved on the second task. Although there were two rating sessions, the rating behavior did not differ between the two sessions (fixed χ^2^= 1.7, *p* = 0.19). However, there was a statistically significant difference in rater leniency when assessing peers vs. assessing themselves. Students were harsher to themselves than to others (fixed χ^2^ = 43.3, *p* = 0.00). In addition to all these, the three speaking standards were found to serve distinctive roles and they were not equal in terms of difficulty (fixed χ^2^ = 20.9, *p* = 0.00). Delivery was slightly more difficult than the other categories with a mean measure of −0.35, while Organization seemed to be the easiest with a mean of 0.30. However, the chi-square statistic is very sensitive to sample size; the actual variation within these facets could be small (Myford and Wolfe, 2004). This will be elaborated on later.

**Table 2:** Summary statistics for all six facets.

	**Facets**					
	**Rater**	**Examinee**	**TaskSession**	**RateSession**	**SelfPeer**	**Standard**
Fixed χ^2^	124.7^*^	201.2^*^	20.1^*^	1.7	43.3^*^	20.9^*^
*p*	0.00	0.00	0.00	0.19	0.00	0.00
*df*	29	28	1	1	2^*^	2
Random χ^2^	24.0	24.8				
*p*	0.68	0.58				
*df*	28	27				
Separation	1.81	2.39				
Strata	2.74	3.52	4.35	0.00	4.81	3.56
Reliability of separation	0.77	0.85	0.90	0.00	0.92	0.85
Measure mean	1.76	0.00	0.00	0.00	0.00	0.00
SD	0.77	0.84	0.28	0.08	0.37	0.27
Infit MnSq mean	1.02	0.99	1.00	0.99	0.97	0.98
SD	0.31	0.27	0.05	0.06	0.08	0.18
Outfit MnSq mean	1.03	1.00	1.01	1.00	0.97	1.00
SD	0.29	0.27	0.07	0.06	0.09	0.17

* *p ≤ 0.05. The degree of freedom is 2 for SelfPeer because there was a teacher rating in the middle (Figure 2)*.

Overall, there were only two unexpected responses (standardized residuals<-3), where rater 3 gave a score lower than expected to examinee 19, and rater 25 gave a lower score to examinee 13 on the delivery dimension. Both scores were for the second speaking task during the second rating session. A quick check on the rater's justification of scores showed that rater 3 believed that examinee 19 tried too hard to pronounce individual words which affected general fluency. Rater 3 also noted stress issues by examinee 19 although he did not explain whether the issue was at the word level or the sentence level. Rater 3 was one of the most stringent raters in this study which might have contributed to this specific low rating. Rater 25 gave example words, such as “*opinion*”, from the recording, and concluded that the speaker mumbled too much on words. What he pointed out was evident in the recording although he forgot that the instruction was to consider comprehensibility rather than pronunciation *per se*. Rater 25 was among the most lenient raters in this study and the overall performance by examinee 13 was above average; thus, this low rating is not reasonable.

### How Do Students Perform in the Process of Peer Assessment?

In general, the results revealed small inter-person variability. For example, the rater separation index was 1.81 for the rater facet with a reliability of 0.77 (Table 2), suggesting that we can reliably separate these raters into about two groups (Eckes, 2009). However, this number was not high, thus, although there was a difference in leniency, the raters were not apples and oranges when awarding scores. The mean item fit statistics also corroborated this conclusion. Both Infit and Outfit means for the rater facet were close to 1 (1.02 and 1.03) with small standard deviations (0.31 and 0.33) (van Moere, 2006). At the individual level, there were four marginally to moderately misfit raters (raters 1, 10, 11, and 20) with Infit larger than 1.5. However, all the corresponding z statistics indicate a good fit (<2). This result was consistent with the previous section where only two unexpected responses were detected across all six facets.

Rater performance in this study can be detected from three more perspectives. Their performance at two rating sessions and on two speaking tasks could testify leniency as a stable or temporary attribute of these students in rating, and the performance during self-assessment vs. peer assessment provides other information, such as social or affective factors in AaL activities.

In general, there was no interaction between facets (Table 3). However, pairwise analyses identified two raters between rating sessions with a biased measurement larger than 1 (1.45 for rater 11 and 1.20 for rater 23). Both raters gave higher scores during the first rating session, but the difference did not reach statistical significance (*t* = 2.08, *p* = 0.07 and *t* = 1.94, *p* = 0.09). For the interaction with the task session, only rater 24 was identified (bias = 1.54). He gave scores higher than expected for the second set of recordings, but the difference still did not reach statistical significance (*t* = 2.17, *p* = 0.08). Thus, the student raters were rather stable in terms of leniency for both rating sessions and when rating recordings of both speaking tasks.

**Table 3:** Bias and interaction summary.

	**Rater by** **RateSession**	**Rater by** **SelfPeer**	**Rater by** **TaskSession**	**Examinee by** **TaskSession**	**Examinee by** **SelfPeer**	**Standard by** **TaskSession**	**Standard by** **RateSession**	**Standard by** **SelfPeer**	**Rater by** **Examinee**
Fixed (all = 0) χ^2^	50.7	38.5	35.7	57.8	78.7	2.3	2.1	5.7	171.7
*df*	60	59	60	58	87	6	6	9	170
*p*	0.80	0.98	0.99	0.48	0.73	0.89	0.91	0.77	0.45
Number of biased cases	2 (Rater 11 and 23)	2 (Rater 6 Self, Rater 10 Self)	1 (Rater 24)	4 (Rater 6, 11, 23, and 19)	7 (5 by the teacher, 2 by peers)	0	0	0	42
Total cases	60	59	60	58	87	6	6	9	170

Regarding the difference between peer assessment vs. self-assessment, the fair average score was 4.20 for the former and 3.86 for the latter compared to the 3.93 of faculty rating (χ^2^ = 43.3, *df* = 2, *p* = 0.00). Pairwise analysis revealed only two individuals with bias (Table 2). Specifically, rater 6 was more lenient to peers than to himself with a marginal bias size of −1.02, and rater 10 was more stringent with peers than with himself with a bias size of 1.20. Both students were below average in terms of their speaking performance although rater 6 was much better than rater 10.

Based on all this, we concluded that after controlling for other facets, rater measures can be used for valid interpretation of assessment behavior in this class. In all, this group of raters performed its duties consistently with each other and was rather lenient in awarding scores. Leniency seemed to be a stable innate feature of this group of college-level advanced learners across time and tasks, although social factors might have played a role which made them more lenient toward others and harsher to themselves in general.

### Results on RQ2: To What Extent Do the Assessment Activities Influence Students' Speaking Performance?

Overall, student ability followed a normal distribution (χ^2^ = 24.8, *df* = 27, *p* = 0.58, Table 2) and can be distinguished into about two band groups (separation = 2.39, reliability = 0.85). This could mean both that raters were able to differentiate quality differences between examinees or that examinees varied in terms of their argumentative speaking abilities. In terms of examinee fit at the individual level, no outlier was detected. In all, evidence supported a good fit of the students' speaking performance as measured by the MFRM model (Linacre, 2018a).

The analyses in the previous section already supported stability in rater leniency, thus the difference in ratings for the first speaking task vs. the second one can be used to testify change in ability. Bias analysis on the interaction between Examinee and TaskSession was not significant in general (χ^2^ = 57.8, *p* = 0.48, Table 3), which corroborated the overall improvement at the group level (χ^2^ = 20.1, *df* = 1, *p* = 0.00) in Table 2.

Pairwise analyses provided some diagnostic information, including four bias terms, all of which were relevant to the second speaking task session. Examinee 19 received a lower score than expected on the second task (biased = −1.11) and examinees 6, 11, and 23 all received higher scores than expected on the second task. However, only the difference involving examinee 23 reached statistical significance (*t* = 2.69, *df* = 8, *p* = 0.03). By checking the recording, we found that his improvement on the second task was rather obvious. Not only did he no longer hesitate as much as he did with the first speaking task, but the content and logic in the second task were also rather good. Thus, the bias statistic in this case was not an issue. This was also acknowledged by the peer rater in her justification for rating on this second task (5 for delivery, 4 for language use, and 5 for organization, respectively):

“*The answer is fluent and the pronunciation is good. The logic of the whole discourse is smooth and the argument is relatively complete. The recording time may be a little bit short. You could use the time to think of and add more and better expressions.”*

In all, statistical evidence supported group-level improvement, and pairwise interaction did not challenge this overall pattern. It is worth emphasizing that the second speaking task every student completed was not of the same topic they were assigned to rate for the first rating session, thus improvement in speaking performance was unlikely to be due to copying verbatim from recordings they had rated in the first round. In conclusion, students' performance improved in the second task.

In terms of the evaluation of rating standards, together with the fixed chi-square (*p* = 0.00), separation (2.42), and reliability (0.85) indices in Table 2, our data seemed to support three distinctive standards, at least in terms of difficulty. Delivery was the most difficult with a fair average score of 3.86, while organization was the easiest with a fair average score of 4.12. Analysis of the interaction between standards with rate session and task session detected no significant result. Thus, the relative difficulty of the three standards was rather stable. In all, students can organize their ideas with relatively smooth logic, but they have more issues with delivery.

### Results on RQ3: What Are Students' Perceptions of the Assessment Activities?

The questionnaire results showed variability in the rating process. Students shared common grounds in rating but also differed in some respects. Table 4 reorganizes the item order and presents the three dimensions of the questionnaire that are relevant to the three research questions in this study. Relevant items in each dimension are presented in the order of mean magnitude. Full descriptive statistics can be found in Appendix B in Supplementary Material.

**Table 4:** Questionnaire results.

**Dimensions**	**Items**	**Mean**	**Median**
Rating attitude	Q21: I think requiring feedback helps with a more accurate and fair assessment.	4.44	5
	Q6: The peer ratings I received are friendly.	3.65	4
	Q2: I like peer-assessment activities.	3.11	3
	Q7: I think the peer ratings I received are more stringent than my self-ratings.	3.08	3.5
	Q1: Peer assessment makes me nervous.	2.59	2
	Q20: Feedback from others helped me know my own strengths and weaknesses.	4.56	5
Rating process	Q19: I think ensuring anonymity in rating is very important.	4.44	5
	Q11: Peer assessment helped me understand the rating standards.	4.19	4
	Q8: I put more effort into rating because of the requirement to justify my decisions and/or provide feedback.	4.15	4
	Q3 I am more cautious when scoring classmates than giving a score to myself.	4.15	4
	Q17: I studied the speaking tasks more in order to complete the peer rating task.	3.96	4
	Q10: The first peer-assessment experience helped with my second speaking task.	3.65	4
	Q13: I checked anything that I was not sure about (such as word usage) in the recordings that I rated.	3.00	3
Rating effects	Q16: This assessment project provided language learning opportunities.	4.19	4
	Q11: Peer assessment helped me understand the rating standards.	4.19	4
	Q14: I found what I needed to improve when I assessed my peer classmates.	4.15	4
	Q22: Self-assessment activities improved my ability to evaluate.	4.15	4
	Q12: Self-assessment helped me understand the rating standards.	3.89	4
	Q23: Peer-assessment activities improved my ability to evaluate.	3.52	4
	Q15: I found what I had done well in speaking when I assessed my peer classmates.	3.37	3

In terms of their attitudes toward PA (see Table 4), most students agreed that the peer ratings they received were fair (mean = 4.44) and friendly (mean = 3.65), and some also expressed their reluctance to be harsh on others. This was consistent with the MFRM results where self-ratings were lower than peer ratings. This theme was also found during the interview, as revealed by the following two excerpts.

Interviewee A: It was a little bit awkward to award scores to my classmates. Although we are given the tasks in anonym, I can still figure out the voice of the recordings, which put a lot of burden on me in exercising scores (code:4-C) (Appendix C in Supplementary Material).
Interviewee B: I found it difficult and embarrassing to give low scores to peers, even though their performances can be really bad sometimes (code:4-A).
But I enjoyed the whole process since it at least gave me opportunities to compare their performance and mine in a private way (code:4-A).

About the rating process, students articulated that the practice of PA helped them clarify the rating standards (mean = 4.19). They also learned to be more responsible when giving scores because of the requirement to provide feedback to justify their ratings (mean = 4.15). They read items more closely (mean = 3.96) and examined the uncertainties when they rated (mean = 3). They admitted that the experience of the first rating process also prepared them well for completing the second speaking task (mean = 3.11).

To look at the rating process in a more detailed way, we found that some raters adjusted their rating strategies after the first session, while some did not.

Interviewee A: I found some significant changes in awarding scores in these two rating sessions. In the first rating procedure, I wasn't prepared, but after I listened to recordings from my peers and received their rating scores and feedback, I tried to make myself fully prepared for my second rating session (code:1-B).
Interviewee B: I didn't find any differences between the first rating session and the second because I still followed the same rating rubrics (code:1-B).

Some of the students also voiced their concerns over challenges in the rating process.

Interviewee C: I found it difficult to distinguish the levels of the categories (code:2-A).
Interviewee A: I think the scale levels need to be more specific to distinguish some features in speaking. There is not enough training time for us to practice rating speech samples (code:2-A).

For the effects of the rating, most students displayed positive attitudes in PA. They argued that through the practice of PA, they detected their weakness (mean=4.15), strength (mean = 3.37), and their ability in evaluation (mean = 4.15). Most importantly, PA provided them with learning opportunities (mean = 4.19).

This was partly evidenced by the interview, amid some mixed feelings.

Interviewee B: The first rating experience helped me with the second because I identified some language use problems of my peers in the first rating process. I also checked the uncertainties in the dictionary during the assessment process (code: 4-A).
Interviewee B: Peer assessment also helped me understand the rating scale more clearly (code: 4-A).
Interviewee C: I learned some good words and expressions from the first rating session that I can use in my second rating session (code: 4-A).
Interviewee D: Rating helped me realize my drawbacks in speaking and helped peers improve their speaking (code: 4-A).
Interviewee A: I didn't find any changes in my rating outcome because I didn't get enough feedback from the peers in the first rating session. Besides, I found some of the peer raters were not professional or qualified enough to award a fair score (codes: 4-B, 4-C).
Interviewee D: I learned from the feedback a lot. It helped me to assess my speaking performance from many different perspectives. However, some feedback is not professional in that they are very vague (codes: 4-A, 4-C).

In all, the questionnaire and interview results were consistent with the MFRM analyses. Students learned from the assessment activities which contributed to the improvement in their speaking performance. There were social factors that interfered with the rating leniency toward peers vs. themselves but this group of students was rather stable in ranking the recordings they received. Thus, these types of assessment activities were feasible in our classroom and were reliable and valid in promoting language learning.

## Discussion

This study examined the effects of PA on students' learning using mixed methods of Rasch measurement, questionnaire, and interview. The findings of this study indicated that the practice of PA provided students with learning opportunities through their involvement in assessments, and they made headways in speaking ability.

### Implications for AaL Research

Our research indicated that students were fully engaged in the speaking assessment and learning, displaying agentic engagement that was also identified by Wang and Lee (2021) in AaL-oriented writing class. Students were placed as critical connectors between assessment and learning through assessment activities. The questionnaire and interview data showed the majority of the students enjoyed the process of PA. The agentic engagement in this study embodied reciprocity in assessment context co-construction, where students were engaged in offering diagnostic peer feedback and seeking clarification on peer feedback. On the other hand, the agentic engagement was also manifested through proactivity in self-regulation. Students employed resourcing strategies (e.g., looking up dictionary), adjusted their speaking strategies, and modified speaking performance based on peer performance. Their second speaking performance and response to the questionnaire and interview manifested a better understanding of the rating criteria. This resonated with previous findings that students can learn to conduct metacognitive operations such as goal setting, goal adjusting, and self-assessment regularly in an AaL context (Lam, 2016; Xiang et al., 2021). The metacognitive experiences they gained in the process of PA enabled them to gradually internalize the speaking criteria and enhance their assessment and feedback literacy.

This study demonstrated the development of students' evaluative judgment, which corroborates the findings in previous AaL research (Boud et al., 2018; Boud, 2022; Xiao and Gu, 2022). In a recent study where Yan and Chuang (2022) followed 30 raters in terms of their growth, the inter-rater agreement rate improved over time. However, the index at the beginning of the study was just 36.7%, which is actually lower than the value in our study (38.2%). Considering the fact that their raters were teachers and graduate students who majored in English in the United States, while our students were non-English freshman students (although from a top university in China), the result is inspiring for AaL research. In all, this study confirmed the feasibility of PA as AaL, which, in turn, supports the validity argument for the interpretation and usage of PA in the classroom.

The practice of PA in this study represented features not only of AaL, but also of PA *per se*. Topping's (2018) theoretical model of PA lent support to the explanations and understandings of the results. The main sub-processes of PA were illustrated in this study as cognitive conflict, scaffolding, and error management and affect. Students were given the opportunities to detect errors in peer spoken performance by using standards. Peers had more time than the teachers and could be qualified to “root out misconceptions” in their partners (Topping, 2018, p. 105). Some students mentioned the benefits of receiving positive feedback and learning useful expressions while assessing peers. Such support and scaffolding, rooted in Vygotsky's (1978) idea of the Zone of Proximal Development (ZPD), do not come from an authoritative teacher but from a competent peer, partner, or classmate. ZPD is described as the distance between an individual's independent functioning performance and the potential performance level with others' assistance. In this study, the improvement of students' speaking in two rating sessions can be interpreted as they reached ZPD through interacting with peers (peer assessment) and themselves (self-assessment). Both these are assessing-as-learning processes and lead to the development of metacognition and self-regulation, which are situated in the deep level of Topping's model (Topping, 2018). In this regard, PA is more than a strategy of evaluative judgment as some AaL research depicted. It can serve as a practical and theoretical tool to interpret the use of AaL.

### Implications for Classroom Teaching

This study dispelled the mistrust of students' capability of being qualified raters (Liu and Carless, 2006) and readiness for peer learning (To and Panadero, 2019). For teachers, this study provides evidence that PA can be regularly exercised in the English classroom, especially where students have moderate to high language proficiency. Together with an appropriate model such as MFRM, which adjusts for measurement errors, student assessment can provide reliable information for decision-making. For students, PA is a good practice for peer learning and provides opportunities to detect their strengths and weakness. Although this study partly confirmed the stress in awarding scores to peers (Wanner and Palmer, 2018), the evidence is more positive than negative. We believe students can practice PA in small groups from time to time when proper guidance is provided.

The ability to assess reflects the ability to understand the criterion and to evaluate one's ability. For example, rater 10's unexpected responses could be driven by a self-rescuing motivation to raise the scores for the course consciously, or it could reflect inaccuracy in self-evaluation. The first interpretation provides information for teachers on class management and score reporting; the latter provides chances for instructional intervention that will benefit student growth. For example, in this study, peer rating was given a small weight as part of the course grade to reduce the stake. The same weight was given to a separate score on the quality of comments that accompanies the rating. This practice might have helped to ensure the high quality of the activity where we only detected one rater whose behavior could not be reasonably justified.

Knowing and detecting the psychology underlying the behavior is part of the assessment literacy for teachers if AaL can function well. The general reluctance to give low scores to peers may reflect a local cultural influence or a universal human nature that teachers need to be aware of. These findings also resonated with previous studies on both EFL writing and speaking where self-raters systematically evaluated their work lower than expected (Matsuno, 2009; Aryadoust, 2015). However, as long as rater leniency remains stable, appropriate statistical modeling can be employed to adjust the scores to overcome this challenge. The complexity of the model, however, might be a barrier to many classroom teachers. In this sense, technical support should be made available and accessible to classroom teachers so that valuable tools can be popularized and not be constrained to researchers only.

Finally, the ability to apply standards is both a result of and the cause of language proficiency. For example, the fact that ratings on the delivery dimension tended to be lower may be due to raters' language competency. Mistakes in pronunciation, pauses, and accents may be easier to detect, and their influence on comprehensibility is more conspicuous and easier to comment on. However, deficiency in organization can be reconciled by background information or examples in the body parts of a discourse. In turn, listening comprehension is not as easily affected. Especially if these elements do not contradict each other, raters may not be as confident in justifying a low score on this dimension as on the delivery dimension. As the assessment ability improves through practice and training, it is likely that raters' language ability and rating performance will also improve.

## Conclusion

This study confirmed the positive effect exerted by PA on students' learning. Our findings demonstrated that students' speaking performance improved across two rating sessions. Surveys and interviews revealed how students learned from peers, internalized the criteria, and adjusted goals and strategies in speaking performance and assessment behavior. By participating in assessment and shouldering the responsibility to help each other, they actively engaged with peers and their learning. The findings add to our understanding of the psychology and behavior in the practice of peer assessment and self-assessment, both of which can be concluded as valuable AaL activities.

The limitations of the study have to be noted. First, students in this study were not engaged in designing and revising standards of the speaking performance. In the future, this can be done so that students may feel more related to and more confident in assessing each other. Second, given that only 29 students in one class were examined, the findings may not be generalizable to a different population in terms of university type, age group, and ethnicity or racial groups. These students are among the top students in China as judged by their college entrance examination scores. Future research can focus on students in other contexts and with various proficiency levels to explore the feasibility and reliability of AaL in different contexts.

## Data Availability Statement

Raw rating data is available upon request from the corresponding author at fchen2020@sjtu.edu.cn.

## Ethics Statement

The studies involving human participants were reviewed and approved by Ethics Committee for Science and Technology of Shanghai Jiao Tong University. The patients/participants provided their written informed consent to participate in this study.

## Author Contributions

SY helped to prepare the speaking prompts, participated in the interviews, took the lead in interview data analyses, and wrote the first drafts. FC designed the complete research and framework, collected the rating data, lead the quantitative data analyses, and finalized all revisions. HC provided curriculum and assessment support, participated in research design, data interpretation, and proofread the manuscripts. All authors contributed to the article and approved the submitted version.

## Conflict of Interest

The authors declare that the research was conducted in the absence of any commercial or financial relationships that could be construed as a potential conflict of interest.

## Publisher's Note

All claims expressed in this article are solely those of the authors and do not necessarily represent those of their affiliated organizations, or those of the publisher, the editors and the reviewers. Any product that may be evaluated in this article, or claim that may be made by its manufacturer, is not guaranteed or endorsed by the publisher.
