# Supplementary material for: Assessment as Learning: How Does Peer Assessment Function in Students' Learning?
Source: Front Psychol. 2022 Jun 27;13:912568. doi: 10.3389/fpsyg.2022.912568 (PMC9271947; doi:10.3389/fpsyg.2022.912568)
Supplement: Supplementary file 1 [file Presentation_1.pdf]

APPENDIX A

Speaking tasks

Instructions: For each question, you have 1 minute to prepare and 2 minutes to respond. We will provide feedback on the qualities of your speech in terms of delivery, language use, topic development (organization, logic, etc.).

Topic 1: Campus life

Question A

It used to be that earning an "A" was enough of a reward for doing well in school. Today, in an effort to boost achievement, some schools give students gift cards or high-tech gadgets. And in a surprising trend, some are rewarding students with cash.

Rewarding students' efforts and achievements with cash is believed to be a positive approach to engaging students in learning. To what extent do you agree or disagree that this is a positive approach?

Question B

An American scientific report found that 70 percent of full-time college students are working part-time nowadays. Previous research has shown that, studying while working can be beneficial for students, though there is a threshold where it starts to do more harm than good.

Do you agree or disagree that the pros of working while in college weigh more than the cons?

Topic 2: Creativity

Question C

It's certainly true that artificial intelligence today can work out the rules as they go along, rather than being taught. AlphaGo, the AI that defeated the Korean go grandmaster Lee Sedol, was fed thousands of games, but no rules. It worked out how to play go entirely by itself. Some argue that this means AI can be more creative than human beings.

Do you think AI can be more creative than human beings?

Question D

Creativity is the ability to use your imagination to produce new ideas or new things. Being creative helps you become a better problem solver in all areas of your life and work. It is regarded as one of the most essential skills in the 21st century.

The issue is, how to promote this skill? Do you think X University should offer courses on creativity?

APPENDIX B

Descriptive statistics of the complete questionnaire

Item	Mean	Median	SD
Q1 Peer assessment makes me nervous.	2.59	2.00	1.19
Q2 I like peer assessment activities.	3.11	3.00	0.80
Q3 I am more cautious when scoring classmates than giving scores to myself.	4.15	4.00	0.77
Q4 It's difficult to use the three standards for scoring.	2.96	3.00	1.06
Q5 My second speaking topic is more difficult than the first one.	2.52	2.00	0.70
Q6 Peer ratings I received are friendly.	3.65	4.00	0.85
Q7 I think the peer ratings I received are more stringent than my self-ratings.	3.08	3.50	1.13
Q8 I put more effort into rating because of the requirement to justify my decisions and/or to provide feedback.	4.15	4.00	0.77
Q9 I tend to give higher scores for longer recordings.	2.81	3.00	1.11
Q10 The first peer assessment experience helped with my second speaking task.	3.65	4.00	1.13
Q11 Peer assessment helped me understand the rating standards.	4.19	4.00	0.56
Q12 Self-assessment helped me understand the rating standards.	3.89	4.00	0.64
Q13 I checked anything that I was not sure about (such as word usage) in the speaking recordings that I rated.	3.00	3.00	1.14
Q14 I found what I needed to improve when I assessed my peer classmates.	4.15	4.00	0.72
Q15 I found what I had done well when I assessed my peer classmates.	3.37	3.00	0.79
Q16 This assessment project provided language learning opportunities.	4.19	4.00	0.62
Q17 I studied the speaking tasks more in order to complete the peer rating task.	3.96	4.00	0.76
Q18 I recognized the voices in the recordings.	3.00	3.00	1.27
Q19 I think ensuring anonymity in rating is very important.	4.44	5.00	0.69
Q20 Feedback from others helped me to know my own strengths and weaknesses.	4.56	5.00	0.51
Q21 I think requiring feedback helps with a more accurate and fair assessment.	4.44	5.00	0.70
Q22 Self-assessment activities improved my ability to evaluate.	4.15	4.00	0.66
Q23 Peer-assessment activities improved my ability to evaluate.	3.52	4.00	0.90

APPENDIX C

Coding scheme of the semi-structured interview

Categories	Sub-categories	Codes 1
1. Strategies involved	A. Listening comprehension strategies	1-A
	B. Rating strategies	1-B
2. On the rating process	A. Difficulty in distinguishing levels	2-A
	B. Workload in rating	2-B
3. On the rating rubric	A. Positive attitude	3-A
	B. Negative attitude	3-B
	C. Mixed	3-C
4. Post-event summary	A. Positive	4-A
	B. Negative	4-B
	C. Mixed	4-C
5. Other comments	A. Suggestions for rating feedback	5-A
	B. Suggestions for rating frequency	5-B
	C. Suggestions for rating procedure	5-C

APPENDIX D

FACETS input

```
Title = MFRM

; 30 raters scored 29 persons' responses to 2 Speaking Tasks.

; This is an incomplete but linked design.

; Every rater rated 6 recordings in total.


facets = 6        ; there are 6 facets in this analysis

noncenter = 1  ; rater facet (facet 1) floats.

positive =1,2,3,4,5,6       ; greater score means greater measure for all facets

Inter-rater = 1  ; facet 1 is the rater facet


Model=

?,?,?,?,?,?,R5             ; No interactions

;?B,?B,?,?,?,?,R5          ; look for interaction/bias between Rater* Examinee

;?B,?,?,?B,?,?,R5          ; look for interaction/bias between Rater* RateSession

;?B,?,?,?,?B,?,R5          ; look for interaction/bias between Rater* SelfPeer

;?B,?,?B,?,?,?,R5          ; look for interaction/bias between Rater * TaskSession


;?,?B,?B,?,?,?,R5          ; look for interaction/bias between Examinee* TaskSession

;?,?B,?,?,?B,?,R5          ; look for interaction/bias between Examinee* SelfPeer


;?,?,?B,?,?,?B,R5          ; look for interaction/bias between Standard* TaskSession

;?,?,?,?B,?,?B,R5          ; look for interaction/bias between Standard* RateSession

;?,?,?,?,?B,?B,R5          ; look for interaction/bias between Standard* SelfPeer


; Vertical=

;1A, 2A, 3A, 4A,5A,6A, #S
```

```
*
Labels=
1, Rater
 1-30        ; 30 anonymous raters
*
2, Examinee
1-29 ; 29 speakers
*
3, TaskSession
1-2
*
4, RateSession
1-2
*
5, SelfPeer
1-3
1, Peer
2, Self
3, Faculty
*
6, Standard
 1, Delivery             ; 3 standards in total
 2, Language Use
 3, Organization
*
DATA=……
*
```
